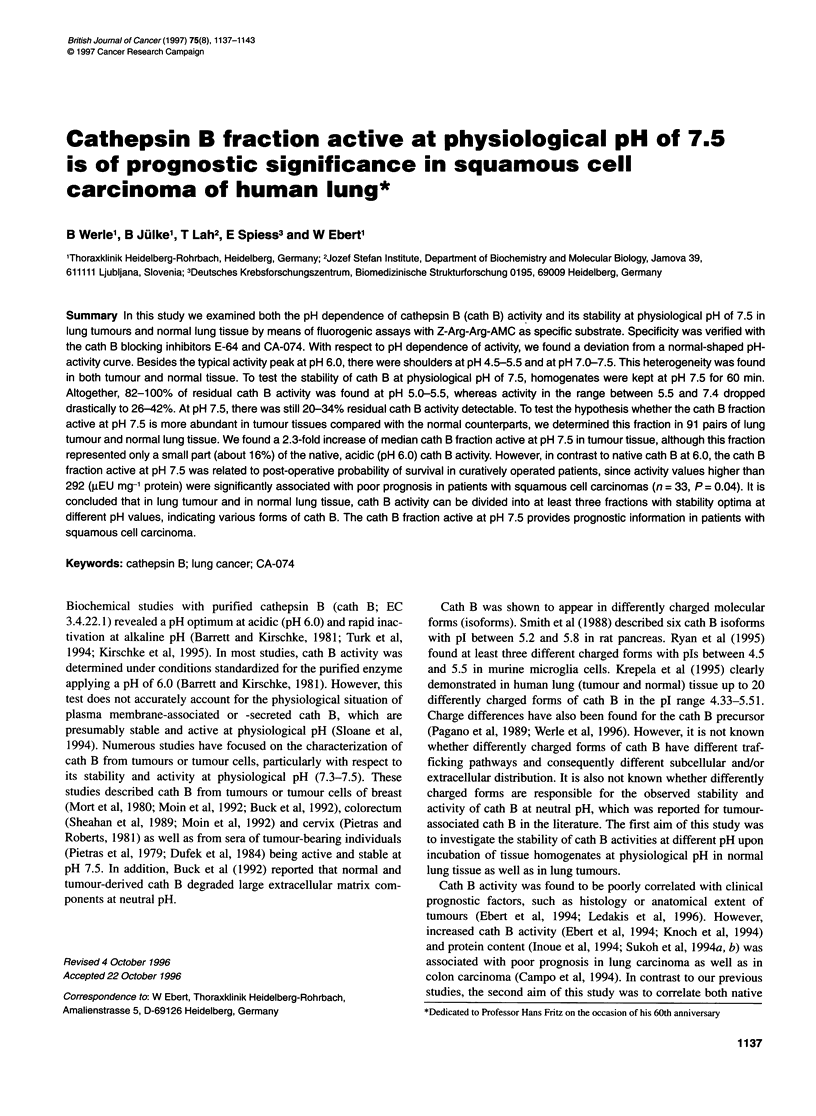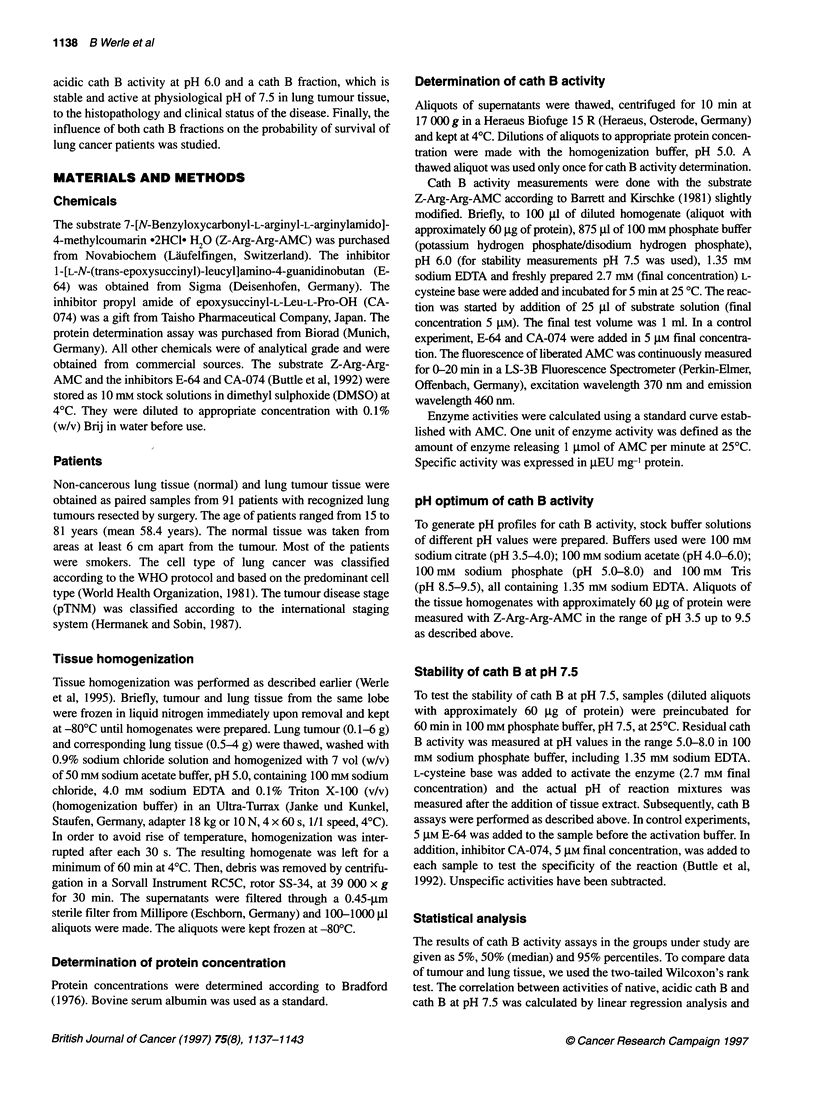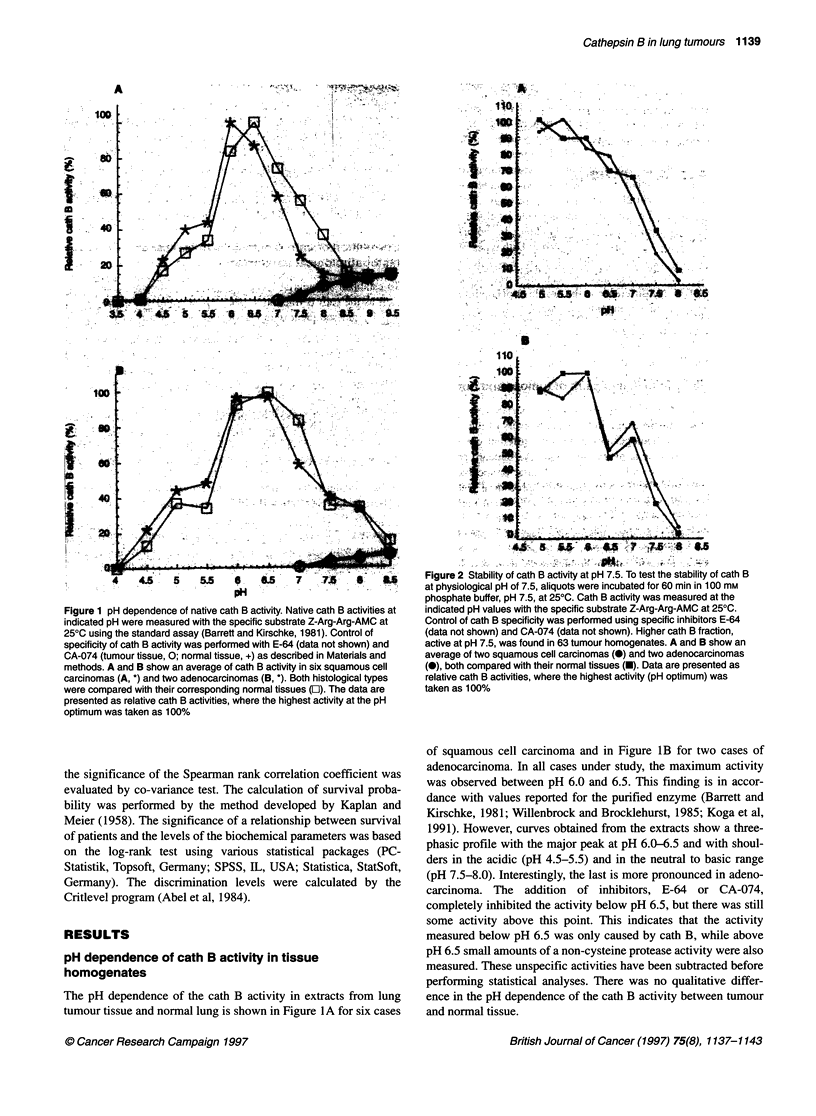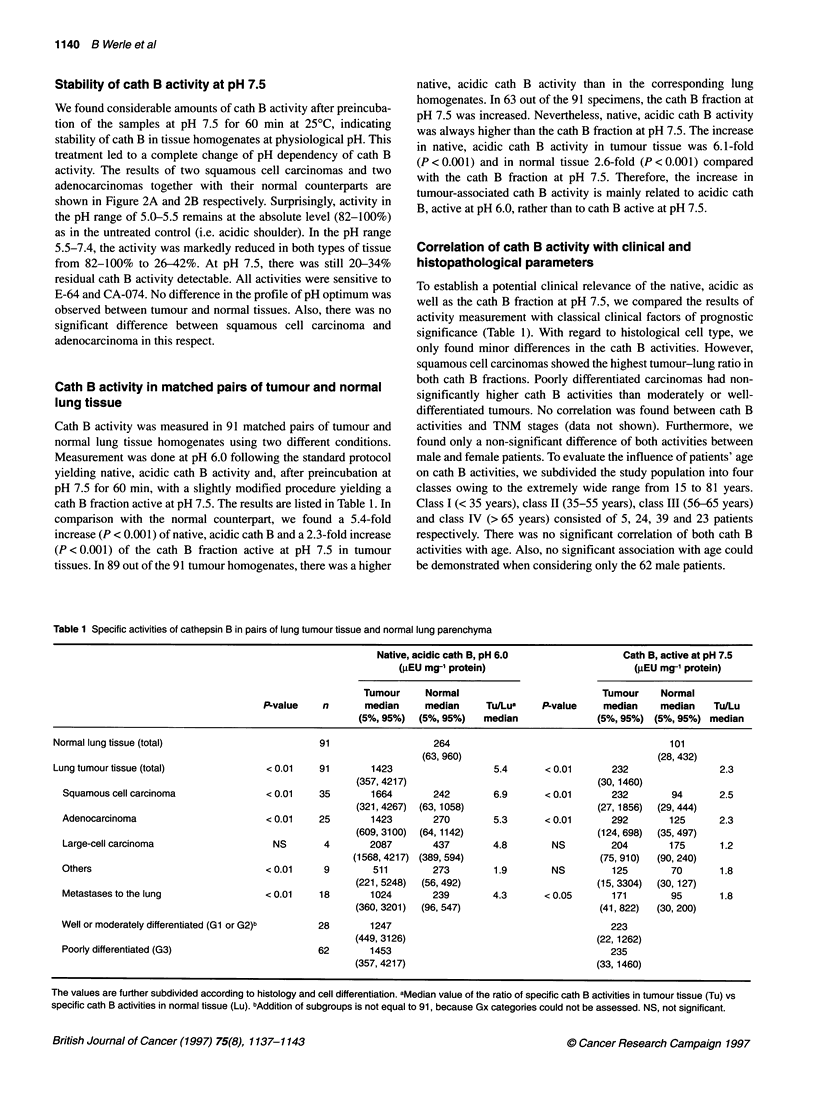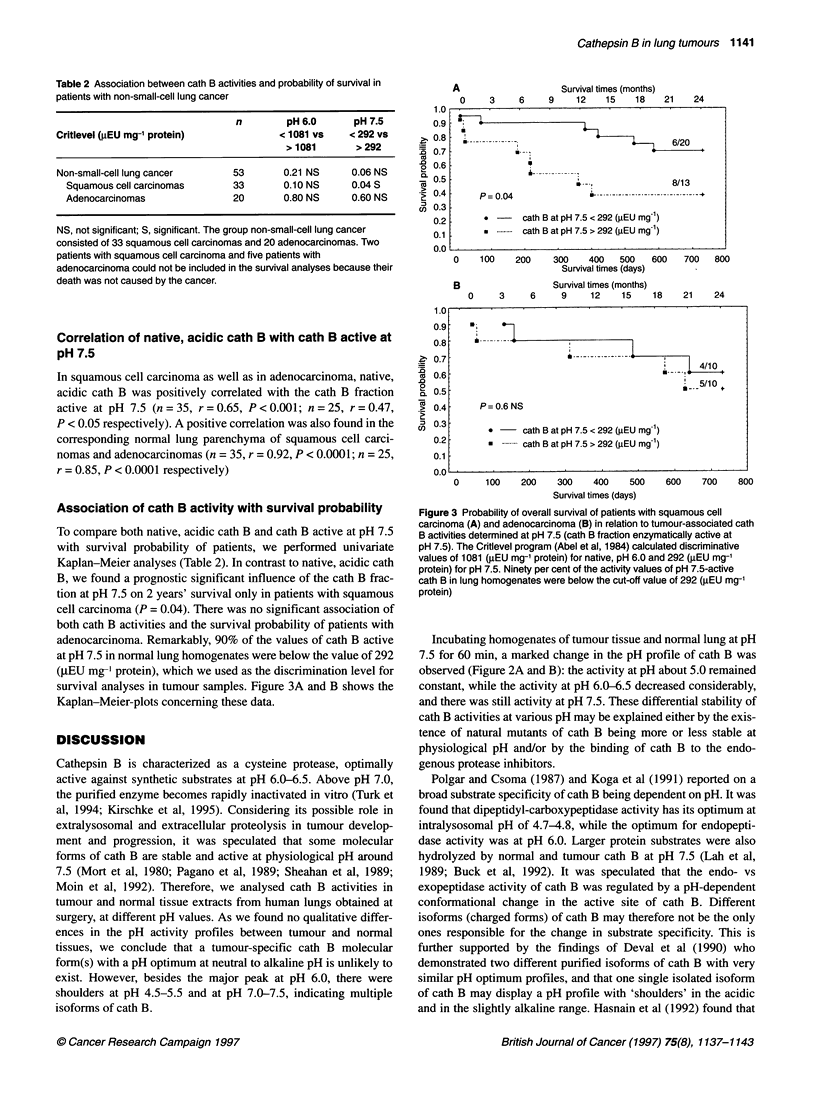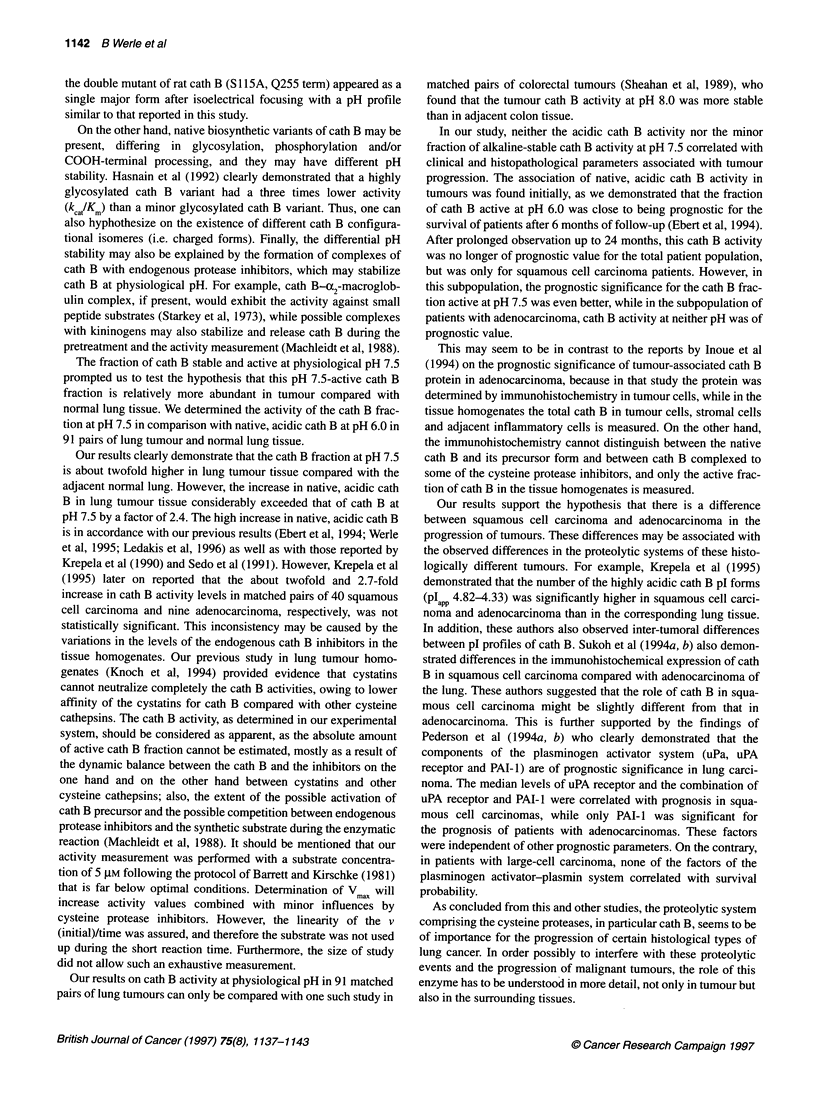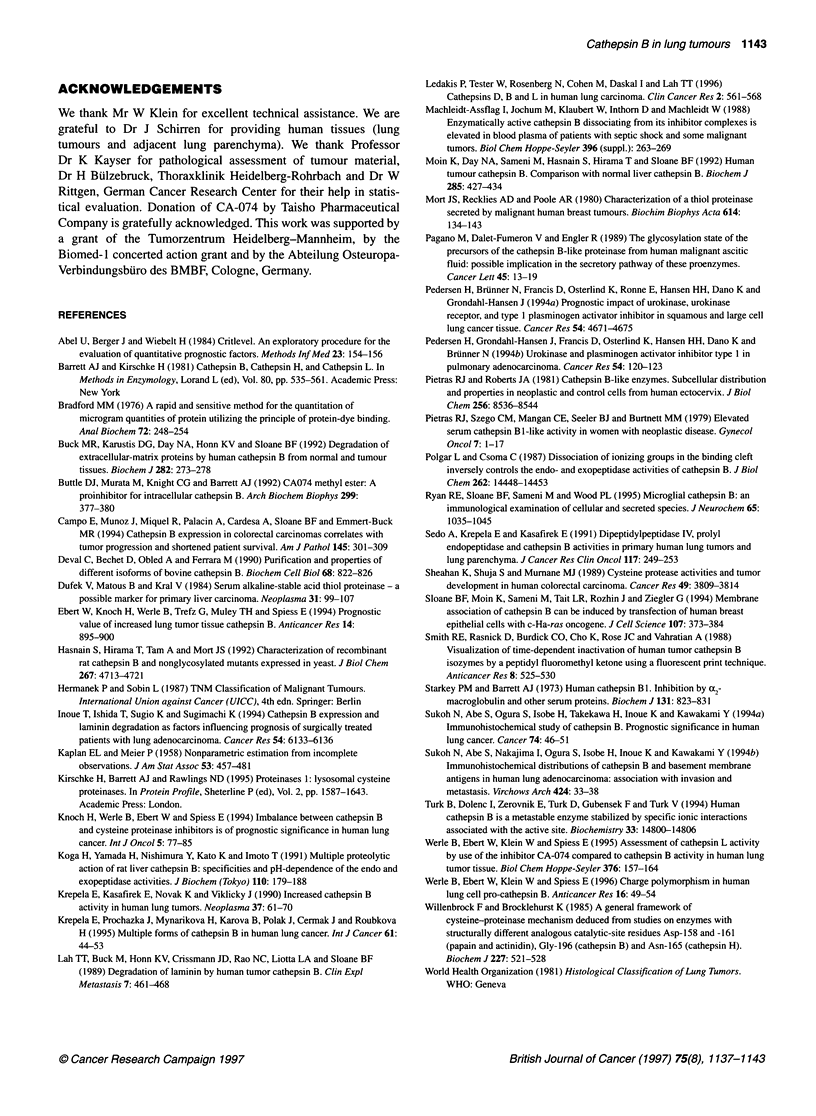# Cathepsin B fraction active at physiological pH of 7.5 is of prognostic significance in squamous cell carcinoma of human lung.

**DOI:** 10.1038/bjc.1997.196

**Published:** 1997

**Authors:** B. Werle, B. JÃ¼lke, T. Lah, E. Spiess, W. Ebert

**Affiliations:** Thoraxklinik Heidelberg-Rohrbach, Heidelberg, Germany.

## Abstract

In this study we examined both the pH dependence of cathepsin B (cath B) activity and its stability at physiological pH of 7.5 in lung tumours and normal lung tissue by means of fluorogenic assays with Z-Arg-Arg-AMC as specific substrate. Specificity was verified with the cath B blocking inhibitors E-64 and CA-074. With respect to pH dependence of activity, we found a deviation from a normal-shaped pH- activity curve. Besides the typical activity peak at pH 6.0, there were shoulders at pH 4.5-5.5 and at pH 7.0-7.5. This heterogeneity was found in both tumour and normal tissue. To test the stability of cath B at physiological pH of 7.5, homogenates were kept at pH 7.5 for 60 min. Altogether, 82-100% of residual cath B activity was found at pH 5.0-5.5, whereas activity in the range between 5.5 and 7.4 dropped drastically to 26-42%. At pH 7.5, there was still 20-34% residual cath B activity detectable. To test the hypothesis whether the cath B fraction active at pH 7.5 is more abundant in tumour tissues compared with the normal counterparts, we determined this fraction in 91 pairs of lung tumour and normal lung tissue. We found a 2.3-fold increase of median cath B fraction active at pH 7.5 in tumour tissue, although this fraction represented only a small part (about 16%) of the native, acidic (pH 6.0) cath B activity. However, in contrast to native cath B at 6.0, the cath B fraction active at pH 7.5 was related to post-operative probability of survival in curatively operated patients, since activity values higher than 292 (muEU mg(-1) protein) were significantly associated with poor prognosis in patients with squamous cell carcinomas (n = 33, P= 0.04). It is concluded that in lung tumour and in normal lung tissue, cath B activity can be divided into at least three fractions with stability optima at different pH values, indicating various forms of cath B. The cath B fraction active at pH 7.5 provides prognostic information in patients with squamous cell carcinoma.


British Journal of Cancer (1997) 75(8), 1137–1143
© 1997 Cancer Research Campaign

### Cathepsin B fraction active at physiological pH of 7.5 is of prognostic significance in squamous cell carcinoma of human lung*

**B Werle[1], B Jülke[1], T Lah[2], E Spiess[3] and W Ebert[1]**

[1]Thoraxklinik Heidelberg-Rohrbach, Heidelberg, Germany; [2]Jozef Stefan Institute, Department of Biochemistry and Molecular Biology, Jamova 39, 611111 Ljubljana, Slovenia; [3]Deutsches Krebsforschungszentrum, Biomedizinische Strukturforschung 0195, 69009 Heidelberg, Germany

**Summary** In this study we examined both the pH dependence of cathepsin B (cath B) activity and its stability at physiological pH of 7.5 in lung tumours and normal lung tissue by means of fluorogenic assays with Z-Arg-Arg-AMC as specific substrate. Specificity was verified with the cath B blocking inhibitors E-64 and CA-074. With respect to pH dependence of activity, we found a deviation from a normal-shaped pH-activity curve. Besides the typical activity peak at pH 6.0, there were shoulders at pH 4.5–5.5 and at pH 7.0–7.5. This heterogeneity was found in both tumour and normal tissue. To test the stability of cath B at physiological pH of 7.5, homogenates were kept at pH 7.5 for 60 min. Altogether, 82–100% of residual cath B activity was found at pH 5.0–5.5, whereas activity in the range between 5.5 and 7.4 dropped drastically to 26–42%. At pH 7.5, there was still 20–34% residual cath B activity detectable. To test the hypothesis whether the cath B fraction active at pH 7.5 is more abundant in tumour tissues compared with the normal counterparts, we determined this fraction in 91 pairs of lung tumour and normal lung tissue. We found a 2.3-fold increase of median cath B fraction active at pH 7.5 in tumour tissue, although this fraction represented only a small part (about 16%) of the native, acidic (pH 6.0) cath B activity. However, in contrast to native cath B at 6.0, the cath B fraction active at pH 7.5 was related to post-operative probability of survival in curatively operated patients, since activity values higher than 292 ($\mu$EU mg$^{-1}$ protein) were significantly associated with poor prognosis in patients with squamous cell carcinomas ($n = 33$, $P = 0.04$). It is concluded that in lung tumour and in normal lung tissue, cath B activity can be divided into at least three fractions with stability optima at different pH values, indicating various forms of cath B. The cath B fraction active at pH 7.5 provides prognostic information in patients with squamous cell carcinoma.

**Keywords:** cathepsin B; lung cancer; CA-074

Biochemical studies with purified cathepsin B (cath B; EC 3.4.22.1) revealed a pH optimum at acidic (pH 6.0) and rapid inactivation at alkaline pH (Barrett and Kirschke, 1981; Turk et al, 1994; Kirschke et al, 1995). In most studies, cath B activity was determined under conditions standardized for the purified enzyme applying a pH of 6.0 (Barrett and Kirschke, 1981). However, this test does not accurately account for the physiological situation of plasma membrane-associated or -secreted cath B, which are presumably stable and active at physiological pH (Sloane et al, 1994). Numerous studies have focused on the characterization of cath B from tumours or tumour cells, particularly with respect to its stability and activity at physiological pH (7.3–7.5). These studies described cath B from tumours or tumour cells of breast (Mort et al, 1980; Moin et al, 1992; Buck et al, 1992), colorectum (Sheahan et al, 1989; Moin et al, 1992) and cervix (Pietras and Roberts, 1981) as well as from sera of tumour-bearing individuals (Pietras et al, 1979; Dufek et al, 1984) being active and stable at pH 7.5. In addition, Buck et al (1992) reported that normal and tumour-derived cath B degraded large extracellular matrix components at neutral pH.

*Revised 4 October 1996*
*Accepted 22 October 1996*

*Correspondence to*: W Ebert, Thoraxklinik Heidelberg-Rohrbach, Amalienstrasse 5, D-69126 Heidelberg, Germany

Cath B was shown to appear in differently charged molecular forms (isoforms). Smith et al (1988) described six cath B isoforms with pI between 5.2 and 5.8 in rat pancreas. Ryan et al (1995) found at least three different charged forms with pIs between 4.5 and 5.5 in murine microglia cells. Krepela et al (1995) clearly demonstrated in human lung (tumour and normal) tissue up to 20 differently charged forms of cath B in the pI range 4.33–5.51. Charge differences have also been found for the cath B precursor (Pagano et al, 1989; Werle et al, 1996). However, it is not known whether differently charged forms of cath B have different trafficking pathways and consequently different subcellular and/or extracellular distribution. It is also not known whether differently charged forms are responsible for the observed stability and activity of cath B at neutral pH, which was reported for tumour-associated cath B in the literature. The first aim of this study was to investigate the stability of cath B activities at different pH upon incubation of tissue homogenates at physiological pH in normal lung tissue as well as in lung tumours.

Cath B activity was found to be poorly correlated with clinical prognostic factors, such as histology or anatomical extent of tumours (Ebert et al, 1994; Ledakis et al, 1996). However, increased cath B activity (Ebert et al, 1994; Knoch et al, 1994) and protein content (Inoue et al, 1994; Sukoh et al, 1994*a, b*) was associated with poor prognosis in lung carcinoma as well as in colon carcinoma (Campo et al, 1994). In contrast to our previous studies, the second aim of this study was to correlate both native

*Dedicated to Professor Hans Fritz on the occasion of his 60th anniversary acidic cath B activity at pH 6.0 and a cath B fraction, which is stable and active at physiological pH of 7.5 in lung tumour tissue, to the histopathology and clinical status of the disease. Finally, the influence of both cath B fractions on the probability of survival of lung cancer patients was studied.

### MATERIALS AND METHODS

### Chemicals

The substrate 7-[*N*-Benzyloxycarbonyl-L-arginyl-L-arginylamido]-4-methylcoumarin •2HCl• $H_2O$ (Z-Arg-Arg-AMC) was purchased from Novabiochem (Läufelfingen, Switzerland). The inhibitor 1-[L-*N*-(trans-epoxysuccinyl)-leucyl]amino-4-guanidinobutan (E-64) was obtained from Sigma (Deisenhofen, Germany). The inhibitor propyl amide of epoxysuccinyl-L-Leu-L-Pro-OH (CA-074) was a gift from Taisho Pharmaceutical Company, Japan. The protein determination assay was purchased from Biorad (Munich, Germany). All other chemicals were of analytical grade and were obtained from commercial sources. The substrate Z-Arg-Arg-AMC and the inhibitors E-64 and CA-074 (Buttle et al, 1992) were stored as 10 mM stock solutions in dimethyl sulphoxide (DMSO) at 4°C. They were diluted to appropriate concentration with 0.1% (w/v) Brij in water before use.

### Patients

Non-cancerous lung tissue (normal) and lung tumour tissue were obtained as paired samples from 91 patients with recognized lung tumours resected by surgery. The age of patients ranged from 15 to 81 years (mean 58.4 years). The normal tissue was taken from areas at least 6 cm apart from the tumour. Most of the patients were smokers. The cell type of lung cancer was classified according to the WHO protocol and based on the predominant cell type (World Health Organization, 1981). The tumour disease stage (pTNM) was classified according to the international staging system (Hermanek and Sobin, 1987).

### Tissue homogenization

Tissue homogenization was performed as described earlier (Werle et al, 1995). Briefly, tumour and lung tissue from the same lobe were frozen in liquid nitrogen immediately upon removal and kept at –80°C until homogenates were prepared. Lung tumour (0.1–6 g) and corresponding lung tissue (0.5–4 g) were thawed, washed with 0.9% sodium chloride solution and homogenized with 7 vol (w/v) of 50 mM sodium acetate buffer, pH 5.0, containing 100 mM sodium chloride, 4.0 mM sodium EDTA and 0.1% Triton X-100 (v/v) (homogenization buffer) in an Ultra-Turrax (Janke und Kunkel, Staufen, Germany, adapter 18 kg or 10 N, 4 × 60 s, 1/1 speed, 4°C). In order to avoid rise of temperature, homogenization was interrupted after each 30 s. The resulting homogenate was left for a minimum of 60 min at 4°C. Then, debris was removed by centrifugation in a Sorvall Instrument RC5C, rotor SS-34, at 39 000 × ***g*** for 30 min. The supernatants were filtered through a 0.45-μm sterile filter from Millipore (Eschborn, Germany) and 100–1000 μl aliquots were made. The aliquots were kept frozen at –80°C.

### Determination of protein concentration

Protein concentrations were determined according to Bradford (1976). Bovine serum albumin was used as a standard.

### Determination of cath B activity

Aliquots of supernatants were thawed, centrifuged for 10 min at 17 000 ***g*** in a Heraeus Biofuge 15 R (Heraeus, Osterode, Germany) and kept at 4°C. Dilutions of aliquots to appropriate protein concentration were made with the homogenization buffer, pH 5.0. A thawed aliquot was used only once for cath B activity determination.

Cath B activity measurements were done with the substrate Z-Arg-Arg-AMC according to Barrett and Kirschke (1981) slightly modified. Briefly, to 100 μl of diluted homogenate (aliquot with approximately 60 μg of protein), 875 μl of 100 mM phosphate buffer (potassium hydrogen phosphate/disodium hydrogen phosphate), pH 6.0 (for stability measurements pH 7.5 was used), 1.35 mM sodium EDTA and freshly prepared 2.7 mM (final concentration) L-cysteine base were added and incubated for 5 min at 25 °C. The reaction was started by addition of 25 μl of substrate solution (final concentration 5 μM). The final test volume was 1 ml. In a control experiment, E-64 and CA-074 were added in 5 μM final concentration. The fluorescence of liberated AMC was continuously measured for 0–20 min in a LS-3B Fluorescence Spectrometer (Perkin-Elmer, Offenbach, Germany), excitation wavelength 370 nm and emission wavelength 460 nm.

Enzyme activities were calculated using a standard curve established with AMC. One unit of enzyme activity was defined as the amount of enzyme releasing 1 μmol of AMC per minute at 25°C. Specific activity was expressed in μEU $mg^{-1}$ protein.

### pH optimum of cath B activity

To generate pH profiles for cath B activity, stock buffer solutions of different pH values were prepared. Buffers used were 100 mM sodium citrate (pH 3.5–4.0); 100 mM sodium acetate (pH 4.0–6.0); 100 mM sodium phosphate (pH 5.0–8.0) and 100 mM Tris (pH 8.5–9.5), all containing 1.35 mM sodium EDTA. Aliquots of the tissue homogenates with approximately 60 μg of protein were measured with Z-Arg-Arg-AMC in the range of pH 3.5 up to 9.5 as described above.

### Stability of cath B at pH 7.5

To test the stability of cath B at pH 7.5, samples (diluted aliquots with approximately 60 μg of protein) were preincubated for 60 min in 100 mM phosphate buffer, pH 7.5, at 25°C. Residual cath B activity was measured at pH values in the range 5.0–8.0 in 100 mM sodium phosphate buffer, including 1.35 mM sodium EDTA. L-cysteine base was added to activate the enzyme (2.7 mM final concentration) and the actual pH of reaction mixtures was measured after the addition of tissue extract. Subsequently, cath B assays were performed as described above. In control experiments, 5 μM E-64 was added to the sample before the activation buffer. In addition, inhibitor CA-074, 5 μM final concentration, was added to each sample to test the specificity of the reaction (Buttle et al, 1992). Unspecific activities have been subtracted.

### Statistical analysis

The results of cath B activity assays in the groups under study are given as 5%, 50% (median) and 95% percentiles. To compare data of tumour and lung tissue, we used the two-tailed Wilcoxon's rank test. The correlation between activities of native, acidic cath B and cath B at pH 7.5 was calculated by linear regression analysis and

© Cancer Research Campaign 1997

**Figure 1** pH dependence of native cath B activity. Native cath B activities at indicated pH were measured with the specific substrate Z-Arg-Arg-AMC at 25°C using the standard assay (Barrett and Kirschke, 1981). Control of specificity of cath B activity was performed with E-64 (data not shown) and CA-074 (tumour tissue, O; normal tissue, +) as described in Materials and methods. **A** and **B** show an average of cath B activity in six squamous cell carcinomas (**A**, *) and two adenocarcinomas (**B**, *). Both histological types were compared with their corresponding normal tissues (□). The data are presented as relative cath B activities, where the highest activity at the pH optimum was taken as 100%

**Figure 2** Stability of cath B activity at pH 7.5. To test the stability of cath B at physiological pH of 7.5, aliquots were incubated for 60 min in 100 mM phosphate buffer, pH 7.5, at 25°C. Cath B activity was measured at the indicated pH values with the specific substrate Z-Arg-Arg-AMC at 25°C. Control of cath B specificity was performed using specific inhibitors E-64 (data not shown) and CA-074 (data not shown). Higher cath B fraction, active at pH 7.5, was found in 63 tumour homogenates. **A** and **B** show an average of two squamous cell carcinomas (●) and two adenocarcinomas (●), both compared with their normal tissues (■). Data are presented as relative cath B activities, where the highest activity (pH optimum) was taken as 100%

the significance of the Spearman rank correlation coefficient was evaluated by co-variance test. The calculation of survival probability was performed by the method developed by Kaplan and Meier (1958). The significance of a relationship between survival of patients and the levels of the biochemical parameters was based on the log-rank test using various statistical packages (PC-Statistik, Topsoft, Germany; SPSS, IL, USA; Statistica, StatSoft, Germany). The discrimination levels were calculated by the Critlevel program (Abel et al, 1984).

### RESULTS

### pH dependence of cath B activity in tissue homogenates

The pH dependence of the cath B activity in extracts from lung tumour tissue and normal lung is shown in Figure 1A for six cases of squamous cell carcinoma and in Figure 1B for two cases of adenocarcinoma. In all cases under study, the maximum activity was observed between pH 6.0 and 6.5. This finding is in accordance with values reported for the purified enzyme (Barrett and Kirschke, 1981; Willenbrock and Brocklehurst, 1985; Koga et al, 1991). However, curves obtained from the extracts show a three-phasic profile with the major peak at pH 6.0–6.5 and with shoulders in the acidic (pH 4.5–5.5) and in the neutral to basic range (pH 7.5–8.0). Interestingly, the last is more pronounced in adenocarcinoma. The addition of inhibitors, E-64 or CA-074, completely inhibited the activity below pH 6.5, but there was still some activity above this point. This indicates that the activity measured below pH 6.5 was only caused by cath B, while above pH 6.5 small amounts of a non-cysteine protease activity were also measured. These unspecific activities have been subtracted before performing statistical analyses. There was no qualitative difference in the pH dependence of the cath B activity between tumour and normal tissue.

© Cancer Research Campaign 1997

### Stability of cath B activity at pH 7.5

We found considerable amounts of cath B activity after preincubation of the samples at pH 7.5 for 60 min at 25°C, indicating stability of cath B in tissue homogenates at physiological pH. This treatment led to a complete change of pH dependency of cath B activity. The results of two squamous cell carcinomas and two adenocarcinomas together with their normal counterparts are shown in Figure 2A and 2B respectively. Surprisingly, activity in the pH range of 5.0–5.5 remains at the absolute level (82–100%) as in the untreated control (i.e. acidic shoulder). In the pH range 5.5–7.4, the activity was markedly reduced in both types of tissue from 82–100% to 26–42%. At pH 7.5, there was still 20–34% residual cath B activity detectable. All activities were sensitive to E-64 and CA-074. No difference in the profile of pH optimum was observed between tumour and normal tissues. Also, there was no significant difference between squamous cell carcinoma and adenocarcinoma in this respect.

### Cath B activity in matched pairs of tumour and normal lung tissue

Cath B activity was measured in 91 matched pairs of tumour and normal lung tissue homogenates using two different conditions. Measurement was done at pH 6.0 following the standard protocol yielding native, acidic cath B activity and, after preincubation at pH 7.5 for 60 min, with a slightly modified procedure yielding a cath B fraction active at pH 7.5. The results are listed in Table 1. In comparison with the normal counterpart, we found a 5.4-fold increase ($P < 0.001$) of native, acidic cath B and a 2.3-fold increase ($P < 0.001$) of the cath B fraction active at pH 7.5 in tumour tissues. In 89 out of the 91 tumour homogenates, there was a higher native, acidic cath B activity than in the corresponding lung homogenates. In 63 out of the 91 specimens, the cath B fraction at pH 7.5 was increased. Nevertheless, native, acidic cath B activity was always higher than the cath B fraction at pH 7.5. The increase in native, acidic cath B activity in tumour tissue was 6.1-fold ($P < 0.001$) and in normal tissue 2.6-fold ($P < 0.001$) compared with the cath B fraction at pH 7.5. Therefore, the increase in tumour-associated cath B activity is mainly related to acidic cath B, active at pH 6.0, rather than to cath B active at pH 7.5.

### Correlation of cath B activity with clinical and histopathological parameters

To establish a potential clinical relevance of the native, acidic as well as the cath B fraction at pH 7.5, we compared the results of activity measurement with classical clinical factors of prognostic significance (Table 1). With regard to histological cell type, we only found minor differences in the cath B activities. However, squamous cell carcinomas showed the highest tumour–lung ratio in both cath B fractions. Poorly differentiated carcinomas had non-significantly higher cath B activities than moderately or well-differentiated tumours. No correlation was found between cath B activities and TNM stages (data not shown). Furthermore, we found only a non-significant difference of both activities between male and female patients. To evaluate the influence of patients' age on cath B activities, we subdivided the study population into four classes owing to the extremely wide range from 15 to 81 years. Class I (< 35 years), class II (35–55 years), class III (56–65 years) and class IV (> 65 years) consisted of 5, 24, 39 and 23 patients respectively. There was no significant correlation of both cath B activities with age. Also, no significant association with age could be demonstrated when considering only the 62 male patients.

**Table 1** Specific activities of cathepsin B in pairs of lung tumour tissue and normal lung parenchyma

| | | | Native, acidic cath B, pH 6.0 (μEU mg⁻¹ protein) | | | | Cath B, active at pH 7.5 (μEU mg⁻¹ protein) | | |
|---|---|---|---|---|---|---|---|---|---|
| | *P*-value | *n* | Tumour median (5%, 95%) | Normal median (5%, 95%) | Tu/Lu[a] median | *P*-value | Tumour median (5%, 95%) | Normal median (5%, 95%) | Tu/Lu median |
| Normal lung tissue (total) | | 91 | | 264 (63, 960) | | | | 101 (28, 432) | |
| Lung tumour tissue (total) | < 0.01 | 91 | 1423 (357, 4217) | | 5.4 | < 0.01 | 232 (30, 1460) | | 2.3 |
| Squamous cell carcinoma | < 0.01 | 35 | 1664 (321, 4267) | 242 (63, 1058) | 6.9 | < 0.01 | 232 (27, 1856) | 94 (29, 444) | 2.5 |
| Adenocarcinoma | < 0.01 | 25 | 1423 (609, 3100) | 270 (64, 1142) | 5.3 | < 0.01 | 292 (124, 698) | 125 (35, 497) | 2.3 |
| Large-cell carcinoma | NS | 4 | 2087 (1568, 4217) | 437 (389, 594) | 4.8 | NS | 204 (75, 910) | 175 (90, 240) | 1.2 |
| Others | < 0.01 | 9 | 511 (221, 5248) | 273 (56, 492) | 1.9 | NS | 125 (15, 3304) | 70 (30, 127) | 1.8 |
| Metastases to the lung | < 0.01 | 18 | 1024 (360, 3201) | 239 (96, 547) | 4.3 | < 0.05 | 171 (41, 822) | 95 (30, 200) | 1.8 |
| Well or moderately differentiated (G1 or G2)[b] | | 28 | 1247 (449, 3126) | | | | 223 (22, 1262) | | |
| Poorly differentiated (G3) | | 62 | 1453 (357, 4217) | | | | 235 (33, 1460) | | |

The values are further subdivided according to histology and cell differentiation. [a]Median value of the ratio of specific cath B activities in tumour tissue (Tu) vs specific cath B activities in normal tissue (Lu). [b]Addition of subgroups is not equal to 91, because Gx categories could not be assessed. NS, not significant.

© Cancer Research Campaign 1997

**Table 2** Association between cath B activities and probability of survival in patients with non-small-cell lung cancer

| Critlevel (μEU mg⁻¹ protein) | n | pH 6.0 < 1081 vs > 1081 | pH 7.5 < 292 vs > 292 |
|---|---|---|---|
| Non-small-cell lung cancer | 53 | 0.21 NS | 0.06 NS |
| Squamous cell carcinomas | 33 | 0.10 NS | 0.04 S |
| Adenocarcinomas | 20 | 0.80 NS | 0.60 NS |

NS, not significant; S, significant. The group non-small-cell lung cancer consisted of 33 squamous cell carcinomas and 20 adenocarcinomas. Two patients with squamous cell carcinoma and five patients with adenocarcinoma could not be included in the survival analyses because their death was not caused by the cancer.

### Correlation of native, acidic cath B with cath B active at pH 7.5

In squamous cell carcinoma as well as in adenocarcinoma, native, acidic cath B was positively correlated with the cath B fraction active at pH 7.5 ($n = 35$, $r = 0.65$, $P < 0.001$; $n = 25$, $r = 0.47$, $P < 0.05$ respectively). A positive correlation was also found in the corresponding normal lung parenchyma of squamous cell carcinomas and adenocarcinomas ($n = 35$, $r = 0.92$, $P < 0.0001$; $n = 25$, $r = 0.85$, $P < 0.0001$ respectively)

### Association of cath B activity with survival probability

To compare both native, acidic cath B and cath B active at pH 7.5 with survival probability of patients, we performed univariate Kaplan–Meier analyses (Table 2). In contrast to native, acidic cath B, we found a prognostic significant influence of the cath B fraction at pH 7.5 on 2 years' survival only in patients with squamous cell carcinoma ($P = 0.04$). There was no significant association of both cath B activities and the survival probability of patients with adenocarcinoma. Remarkably, 90% of the values of cath B active at pH 7.5 in normal lung homogenates were below the value of 292 (μEU mg⁻¹ protein), which we used as the discrimination level for survival analyses in tumour samples. Figure 3A and B shows the Kaplan–Meier-plots concerning these data.

### DISCUSSION

Cathepsin B is characterized as a cysteine protease, optimally active against synthetic substrates at pH 6.0–6.5. Above pH 7.0, the purified enzyme becomes rapidly inactivated in vitro (Turk et al, 1994; Kirschke et al, 1995). Considering its possible role in extralysosomal and extracellular proteolysis in tumour development and progression, it was speculated that some molecular forms of cath B are stable and active at physiological pH around 7.5 (Mort et al, 1980; Pagano et al, 1989; Sheahan et al, 1989; Moin et al, 1992). Therefore, we analysed cath B activities in tumour and normal tissue extracts from human lungs obtained at surgery, at different pH values. As we found no qualitative differences in the pH activity profiles between tumour and normal tissues, we conclude that a tumour-specific cath B molecular form(s) with a pH optimum at neutral to alkaline pH is unlikely to exist. However, besides the major peak at pH 6.0, there were shoulders at pH 4.5–5.5 and at pH 7.0–7.5, indicating multiple isoforms of cath B.

**Figure 3** Probability of overall survival of patients with squamous cell carcinoma (**A**) and adenocarcinoma (**B**) in relation to tumour-associated cath B activities determined at pH 7.5 (cath B fraction enzymatically active at pH 7.5). The Critlevel program (Abel et al, 1984) calculated discriminative values of 1081 (μEU mg⁻¹ protein) for native, pH 6.0 and 292 (μEU mg⁻¹ protein) for pH 7.5. Ninety per cent of the activity values of pH 7.5-active cath B in lung homogenates were below the cut-off value of 292 (μEU mg⁻¹ protein)

Incubating homogenates of tumour tissue and normal lung at pH 7.5 for 60 min, a marked change in the pH profile of cath B was observed (Figure 2A and B): the activity at pH about 5.0 remained constant, while the activity at pH 6.0–6.5 decreased considerably, and there was still activity at pH 7.5. These differential stability of cath B activities at various pH may be explained either by the existence of natural mutants of cath B being more or less stable at physiological pH and/or by the binding of cath B to the endogenous protease inhibitors.

Polgar and Csoma (1987) and Koga et al (1991) reported on a broad substrate specificity of cath B being dependent on pH. It was found that dipeptidyl-carboxypeptidase activity has its optimum at intralysosomal pH of 4.7–4.8, while the optimum for endopeptidase activity was at pH 6.0. Larger protein substrates were also hydrolyzed by normal and tumour cath B at pH 7.5 (Lah et al, 1989; Buck et al, 1992). It was speculated that the endo- vs exopeptidase activity of cath B was regulated by a pH-dependent conformational change in the active site of cath B. Different isoforms (charged forms) of cath B may therefore not be the only ones responsible for the change in substrate specificity. This is further supported by the findings of Deval et al (1990) who demonstrated two different purified isoforms of cath B with very similar pH optimum profiles, and that one single isolated isoform of cath B may display a pH profile with 'shoulders' in the acidic and in the slightly alkaline range. Hasnain et al (1992) found that

© Cancer Research Campaign 1997

the double mutant of rat cath B (S115A, Q255 term) appeared as a single major form after isoelectrical focusing with a pH profile similar to that reported in this study.

On the other hand, native biosynthetic variants of cath B may be present, differing in glycosylation, phosphorylation and/or COOH-terminal processing, and they may have different pH stability. Hasnain et al (1992) clearly demonstrated that a highly glycosylated cath B variant had a three times lower activity ($k_{cat}/K_m$) than a minor glycosylated cath B variant. Thus, one can also hyphothesize on the existence of different cath B configurational isomeres (i.e. charged forms). Finally, the differential pH stability may also be explained by the formation of complexes of cath B with endogenous protease inhibitors, which may stabilize cath B at physiological pH. For example, cath B–$\alpha_2$-macroglobulin complex, if present, would exhibit the activity against small peptide substrates (Starkey et al, 1973), while possible complexes with kininogens may also stabilize and release cath B during the pretreatment and the activity measurement (Machleidt et al, 1988).

The fraction of cath B stable and active at physiological pH 7.5 prompted us to test the hypothesis that this pH 7.5-active cath B fraction is relatively more abundant in tumour compared with normal lung tissue. We determined the activity of the cath B fraction at pH 7.5 in comparison with native, acidic cath B at pH 6.0 in 91 pairs of lung tumour and normal lung tissue.

Our results clearly demonstrate that the cath B fraction at pH 7.5 is about twofold higher in lung tumour tissue compared with the adjacent normal lung. However, the increase in native, acidic cath B in lung tumour tissue considerably exceeded that of cath B at pH 7.5 by a factor of 2.4. The high increase in native, acidic cath B is in accordance with our previous results (Ebert et al, 1994; Werle et al, 1995; Ledakis et al, 1996) as well as with those reported by Krepela et al (1990) and Sedo et al (1991). However, Krepela et al (1995) later on reported that the about twofold and 2.7-fold increase in cath B activity levels in matched pairs of 40 squamous cell carcinoma and nine adenocarcinoma, respectively, was not statistically significant. This inconsistency may be caused by the variations in the levels of the endogenous cath B inhibitors in the tissue homogenates. Our previous study in lung tumour homogenates (Knoch et al, 1994) provided evidence that cystatins cannot neutralize completely the cath B activities, owing to lower affinity of the cystatins for cath B compared with other cysteine cathepsins. The cath B activity, as determined in our experimental system, should be considered as apparent, as the absolute amount of active cath B fraction cannot be estimated, mostly as a result of the dynamic balance between the cath B and the inhibitors on the one hand and on the other hand between cystatins and other cysteine cathepsins; also, the extent of the possible activation of cath B precursor and the possible competition between endogenous protease inhibitors and the synthetic substrate during the enzymatic reaction (Machleidt et al, 1988). It should be mentioned that our activity measurement was performed with a substrate concentration of 5 μM following the protocol of Barrett and Kirschke (1981) that is far below optimal conditions. Determination of $V_{max}$ will increase activity values combined with minor influences by cysteine protease inhibitors. However, the linearity of the *v* (initial)/time was assured, and therefore the substrate was not used up during the short reaction time. Furthermore, the size of study did not allow such an exhaustive measurement.

Our results on cath B activity at physiological pH in 91 matched pairs of lung tumours can only be compared with one such study in matched pairs of colorectal tumours (Sheahan et al, 1989), who found that the tumour cath B activity at pH 8.0 was more stable than in adjacent colon tissue.

In our study, neither the acidic cath B activity nor the minor fraction of alkaline-stable cath B activity at pH 7.5 correlated with clinical and histopathological parameters associated with tumour progression. The association of native, acidic cath B activity in tumours was found initially, as we demonstrated that the fraction of cath B active at pH 6.0 was close to being prognostic for the survival of patients after 6 months of follow-up (Ebert et al, 1994). After prolonged observation up to 24 months, this cath B activity was no longer of prognostic value for the total patient population, but was only for squamous cell carcinoma patients. However, in this subpopulation, the prognostic significance for the cath B fraction active at pH 7.5 was even better, while in the subpopulation of patients with adenocarcinoma, cath B activity at neither pH was of prognostic value.

This may seem to be in contrast to the reports by Inoue et al (1994) on the prognostic significance of tumour-associated cath B protein in adenocarcinoma, because in that study the protein was determined by immunohistochemistry in tumour cells, while in the tissue homogenates the total cath B in tumour cells, stromal cells and adjacent inflammatory cells is measured. On the other hand, the immunohistochemistry cannot distinguish between the native cath B and its precursor form and between cath B complexed to some of the cysteine protease inhibitors, and only the active fraction of cath B in the tissue homogenates is measured.

Our results support the hypothesis that there is a difference between squamous cell carcinoma and adenocarcinoma in the progression of tumours. These differences may be associated with the observed differences in the proteolytic systems of these histologically different tumours. For example, Krepela et al (1995) demonstrated that the number of the highly acidic cath B pI forms ($pI_{app}$ 4.82–4.33) was significantly higher in squamous cell carcinoma and adenocarcinoma than in the corresponding lung tissue. In addition, these authors also observed inter-tumoral differences between pI profiles of cath B. Sukoh et al (1994*a*, *b*) also demonstrated differences in the immunohistochemical expression of cath B in squamous cell carcinoma compared with adenocarcinoma of the lung. These authors suggested that the role of cath B in squamous cell carcinoma might be slightly different from that in adenocarcinoma. This is further supported by the findings of Pederson et al (1994*a*, *b*) who clearly demonstrated that the components of the plasminogen activator system (uPa, uPA receptor and PAI-1) are of prognostic significance in lung carcinoma. The median levels of uPA receptor and the combination of uPA receptor and PAI-1 were correlated with prognosis in squamous cell carcinomas, while only PAI-1 was significant for the prognosis of patients with adenocarcinomas. These factors were independent of other prognostic parameters. On the contrary, in patients with large-cell carcinoma, none of the factors of the plasminogen activator–plasmin system correlated with survival probability.

As concluded from this and other studies, the proteolytic system comprising the cysteine proteases, in particular cath B, seems to be of importance for the progression of certain histological types of lung cancer. In order possibly to interfere with these proteolytic events and the progression of malignant tumours, the role of this enzyme has to be understood in more detail, not only in tumour but also in the surrounding tissues.

© Cancer Research Campaign 1997

### ACKNOWLEDGEMENTS

We thank Mr W Klein for excellent technical assistance. We are grateful to Dr J Schirren for providing human tissues (lung tumours and adjacent lung parenchyma). We thank Professor Dr K Kayser for pathological assessment of tumour material, Dr H Bülzebruck, Thoraxklinik Heidelberg-Rohrbach and Dr W Rittgen, German Cancer Research Center for their help in statistical evaluation. Donation of CA-074 by Taisho Pharmaceutical Company is gratefully acknowledged. This work was supported by a grant of the Tumorzentrum Heidelberg–Mannheim, by the Biomed-1 concerted action grant and by the Abteilung Osteuropa-Verbindungsbüro des BMBF, Cologne, Germany.

### REFERENCES

Abel U, Berger J and Wiebelt H (1984) Critlevel. An exploratory procedure for the evaluation of quantitative prognostic factors. *Methods Inf Med* **23**: 154–156

Barrett AJ and Kirschke H (1981) Cathepsin B, Cathepsin H, and Cathepsin L. In *Methods in Enzymology*, Lorand L (ed), Vol. 80, pp. 535–561. Academic Press: New York

Bradford MM (1976) A rapid and sensitive method for the quantitation of microgram quantities of protein utilizing the principle of protein-dye binding. *Anal Biochem* **72**: 248–254

Buck MR, Karustis DG, Day NA, Honn KV and Sloane BF (1992) Degradation of extracellular-matrix proteins by human cathepsin B from normal and tumour tissues. *Biochem J* **282**: 273–278

Buttle DJ, Murata M, Knight CG and Barrett AJ (1992) CA074 methyl ester: A proinhibitor for intracellular cathepsin B. *Arch Biochem Biophys* **299**: 377–380

Campo E, Munoz J, Miquel R, Palacin A, Cardesa A, Sloane BF and Emmert-Buck MR (1994) Cathepsin B expression in colorectal carcinomas correlates with tumor progression and shortened patient survival. *Am J Pathol* **145**: 301–309

Deval C, Bechet D, Obled A and Ferrara M (1990) Purification and properties of different isoforms of bovine cathepsin B. *Biochem Cell Biol* **68**: 822–826

Dufek V, Matous B and Kral V (1984) Serum alkaline-stable acid thiol proteinase – a possible marker for primary liver carcinoma. *Neoplasma* **31**: 99–107

Ebert W, Knoch H, Werle B, Trefz G, Muley TH and Spiess E (1994) Prognostic value of increased lung tumor tissue cathepsin B. *Anticancer Res* **14**: 895–900

Hasnain S, Hirama T, Tam A and Mort JS (1992) Characterization of recombinant rat cathepsin B and nonglycosylated mutants expressed in yeast. *J Biol Chem* **267**: 4713–4721

Hermanek P and Sobin L (1987) TNM Classification of Malignant Tumours. *International Union against Cancer (UICC)*, 4th edn. Springer: Berlin

Inoue T, Ishida T, Sugio K and Sugimachi K (1994) Cathepsin B expression and laminin degradation as factors influencing prognosis of surgically treated patients with lung adenocarcinoma. *Cancer Res* **54**: 6133–6136

Kaplan EL and Meier P (1958) Nonparametric estimation from incomplete observations. *J Am Stat Assoc* **53**: 457–481

Kirschke H, Barrett AJ and Rawlings ND (1995) Proteinases 1: lysosomal cysteine proteinases. In *Protein Profile*, Sheterline P (ed), Vol. 2, pp. 1587–1643. Academic Press: London.

Knoch H, Werle B, Ebert W and Spiess E (1994) Imbalance between cathepsin B and cysteine proteinase inhibitors is of prognostic significance in human lung cancer. *Int J Oncol* **5**: 77–85

Koga H, Yamada H, Nishimura Y, Kato K and Imoto T (1991) Multiple proteolytic action of rat liver cathepsin B: specificities and pH-dependence of the endo and exopeptidase activities. *J Biochem (Tokyo)* **110**: 179–188

Krepela E, Kasafirek E, Novak K and Viklicky J (1990) Increased cathepsin B activity in human lung tumors. *Neoplasma* **37**: 61–70

Krepela E, Prochazka J, Mynarikova H, Karova B, Polak J, Cermak J and Roubkova H (1995) Multiple forms of cathepsin B in human lung cancer. *Int J Cancer* **61**: 44–53

Lah TT, Buck M, Honn KV, Crissmann JD, Rao NC, Liotta LA and Sloane BF (1989) Degradation of laminin by human tumor cathepsin B. *Clin Expl Metastasis* **7**: 461–468

Ledakis P, Tester W, Rosenberg N, Cohen M, Daskal I and Lah TT (1996) Cathepsins D, B and L in human lung carcinoma. *Clin Cancer Res* **2**: 561–568

Machleidt-Assflag I, Jochum M, Klaubert W, Inthorn D and Machleidt W (1988) Enzymatically active cathepsin B dissociating from its inhibitor complexes is elevated in blood plasma of patients with septic shock and some malignant tumors. *Biol Chem Hoppe-Seyler* **396** (suppl.): 263–269

Moin K, Day NA, Sameni M, Hasnain S, Hirama T and Sloane BF (1992) Human tumour cathepsin B. Comparison with normal liver cathepsin B. *Biochem J* **285**: 427–434

Mort JS, Recklies AD and Poole AR (1980) Characterization of a thiol proteinase secreted by malignant human breast tumours. *Biochim Biophys Acta* **614**: 134–143

Pagano M, Dalet-Fumeron V and Engler R (1989) The glycosylation state of the precursors of the cathepsin B-like proteinase from human malignant ascitic fluid: possible implication in the secretory pathway of these proenzymes. *Cancer Lett* **45**: 13–19

Pedersen H, Brünner N, Francis D, Osterlind K, Ronne E, Hansen HH, Dano K and Grondahl-Hansen J (1994*a*) Prognostic impact of urokinase, urokinase receptor, and type 1 plasminogen activator inhibitor in squamous and large cell lung cancer tissue. *Cancer Res* **54**: 4671–4675

Pedersen H, Grondahl-Hansen J, Francis D, Osterlind K, Hansen HH, Dano K and Brünner N (1994*b*) Urokinase and plasminogen activator inhibitor type 1 in pulmonary adenocarcinoma. *Cancer Res* **54**: 120–123

Pietras RJ and Roberts JA (1981) Cathepsin B-like enzymes. Subcellular distribution and properties in neoplastic and control cells from human ectocervix. *J Biol Chem* **256**: 8536–8544

Pietras RJ, Szego CM, Mangan CE, Seeler BJ and Burtnett MM (1979) Elevated serum cathepsin B1-like activity in women with neoplastic disease. *Gynecol Oncol* **7**: 1–17

Polgar L and Csoma C (1987) Dissociation of ionizing groups in the binding cleft inversely controls the endo- and exopeptidase activities of cathepsin B. *J Biol Chem* **262**: 14448–14453

Ryan RE, Sloane BF, Sameni M and Wood PL (1995) Microglial cathepsin B: an immunological examination of cellular and secreted species. *J Neurochem* **65**: 1035–1045

Sedo A, Krepela E and Kasafirek E (1991) Dipeptidylpeptidase IV, prolyl endopeptidase and cathepsin B activities in primary human lung tumors and lung parenchyma. *J Cancer Res Clin Oncol* **117**: 249–253

Sheahan K, Shuja S and Murnane MJ (1989) Cysteine protease activities and tumor development in human colorectal carcinoma. *Cancer Res* **49**: 3809–3814

Sloane BF, Moin K, Sameni M, Tait LR, Rozhin J and Ziegler G (1994) Membrane association of cathepsin B can be induced by transfection of human breast epithelial cells with c-Ha-*ras* oncogene. *J Cell Science* **107**: 373–384

Smith RE, Rasnick D, Burdick CO, Cho K, Rose JC and Vahratian A (1988) Visualization of time-dependent inactivation of human tumor cathepsin B isozymes by a peptidyl fluoromethyl ketone using a fluorescent print technique. *Anticancer Res* **8**: 525–530

Starkey PM and Barrett AJ (1973) Human cathepsin B1. Inhibition by $\alpha_2$-macroglobulin and other serum proteins. *Biochem J* **131**: 823–831

Sukoh N, Abe S, Ogura S, Isobe H, Takekawa H, Inoue K and Kawakami Y (1994*a*) Immunohistochemical study of cathepsin B. Prognostic significance in human lung cancer. *Cancer* **74**: 46–51

Sukoh N, Abe S, Nakajima I, Ogura S, Isobe H, Inoue K and Kawakami Y (1994*b*) Immunohistochemical distributions of cathepsin B and basement membrane antigens in human lung adenocarcinoma: association with invasion and metastasis. *Virchows Arch* **424**: 33–38

Turk B, Dolenc I, Zerovnik E, Turk D, Gubensek F and Turk V (1994) Human cathepsin B is a metastable enzyme stabilized by specific ionic interactions associated with the active site. *Biochemistry* **33**: 14800–14806

Werle B, Ebert W, Klein W and Spiess E (1995) Assessment of cathepsin L activity by use of the inhibitor CA-074 compared to cathepsin B activity in human lung tumor tissue. *Biol Chem Hoppe-Seyler* **376**: 157–164

Werle B, Ebert W, Klein W and Spiess E (1996) Charge polymorphism in human lung cell pro-cathepsin B. *Anticancer Res* **16**: 49–54

Willenbrock F and Brocklehurst K (1985) A general framework of cysteine–proteinase mechanism deduced from studies on enzymes with structurally different analogous catalytic-site residues Asp-158 and -161 (papain and actinidin), Gly-196 (cathepsin B) and Asn-165 (cathepsin H). *Biochem J* **227**: 521–528

World Health Organization (1981) *Histological Classification of Lung Tumors*. WHO: Geneva

© Cancer Research Campaign 1997